# The impact of amplification on differential expression analyses by RNA-seq

**DOI:** 10.1038/srep25533

**Published:** 2016-05-09

**Authors:** Swati Parekh, Christoph Ziegenhain, Beate Vieth, Wolfgang Enard, Ines Hellmann

**Affiliations:** 1 Anthropology & Human Genomics, Department of Biology II, Ludwig-Maximilians University, Großhaderner Str. 2, 82152 Martinsried, Germany

## Abstract

Currently, quantitative RNA-seq methods are pushed to work with increasingly small starting amounts of RNA that require amplification. However, it is unclear how much noise or bias amplification introduces and how this affects precision and accuracy of RNA quantification. To assess the effects of amplification, reads that originated from the same RNA molecule (PCR-duplicates) need to be identified. Computationally, read duplicates are defined by their mapping position, which does not distinguish PCR- from natural duplicates and hence it is unclear how to treat duplicated reads. Here, we generate and analyse RNA-seq data sets prepared using three different protocols (Smart-Seq, TruSeq and UMI-seq). We find that a large fraction of computationally identified read duplicates are not PCR duplicates and can be explained by sampling and fragmentation bias. Consequently, the computational removal of duplicates does improve neither accuracy nor precision and can actually worsen the power and the False Discovery Rate (FDR) for differential gene expression. Even when duplicates are experimentally identified by unique molecular identifiers (UMIs), power and FDR are only mildly improved. However, the pooling of samples as made possible by the early barcoding of the UMI-protocol leads to an appreciable increase in the power to detect differentially expressed genes.

High throughput RNA sequencing methods (RNA-seq) are currently replacing microarrays as the method of choice for gene expression quantification^1^,^2^,^3^,^4^,^5^. For many applications RNA-seq technologies are required to become more sensitive, the goal being to detect rare transcripts in single cells. However, sensitivity, accuracy and precision of transcript quantification strongly depend on how the mRNA is converted into the cDNA that is eventually sequenced^6^. Especially when starting from low amounts of RNA, amplification is necessary to generate enough cDNA for sequencing^7^,^8^. While it is known that PCR does not amplify all sequences equally well^9^,^10^,^11^, PCR amplification is used in popular RNA-seq library preparation protocols such as TruSeq or Smart-Seq^12^. However, it is unclear how PCR bias affects quantitative RNA-seq analyses and to what extent PCR amplification adds noise and hence reduces the precision of transcript quantification. For detecting differentially expressed genes this is even more important than accuracy because it influences the power and potentially the false discovery rate.

RNA-seq library preparation methods are designed with different goals in mind. TruSeq is a method of choice, if there is sufficient starting material, while the Smart-Seq protocol is better suited for low starting amounts^13^,^14^. Furthermore, methods using UMIs and cellular barcodes have been optimized for low starting amounts and low costs, to generate RNA-seq profiles from single cells^7^,^15^. To achieve these goals, the methods differ in a number of steps that will also impact the probability of read duplicates and their detection (Fig. 1). TruSeq uses heat-fragmentation of mRNA and the only amplification is the amplification of the sequencing library. Thus all PCR duplicates can be identified by their mapping positions. In contrast, in the Smart-Seq protocol full length mRNAs are reverse transcribed, pre-amplified and the amplified cDNA is then fragmented with a Tn5 transposase^12^. Consequently, PCR duplicates that arise during the pre-amplification step can not be identified by their mapping positions. UMI-seq also amplifies full-length cDNA, but unique molecular identifiers (UMIs) as well as library barcodes are already introduced during reverse transcription before pre-amplification^16^. This early barcoding allows all samples to be pooled right after reverse transcription. The primer sequences required for the library amplification are introduced at the 3′ end during reverse transcription. Thus, PCR-duplicates in UMI-seq data can always be identified via the UMI. In summary, while PCR-duplicates can be unambiguously identified in UMI-seq, for Smart-Seq and TruSeq PCR-duplicates are identified computationally as read duplicates. However, such read duplicates can also arise by sampling independent molecules. The chance that such natural duplicates, i.e. read duplicates that originated from different mRNA molecules, occur for a transcript of a given length, increases with expression levels and fragmentation bias.

That said, it is unclear whether removing read duplicates computationally improves accuracy and precision by reducing PCR bias and noise or whether it decreases accuracy and precision by removing genuine information. Here, we investigate the impact of PCR amplification on RNA-seq by analyzing datasets prepared with Smart-Seq, TruSeq and UMI-seq as well as different amounts of amplification. We investigate the source of read duplicates by analysing PCR bias and fragmentation bias, assess the accuracy using ERCCs - spike-in mRNAs of known concentrations^17^,^18^ - and assess precision using power simulations using PROPER^19^.

## Results

### Selection of datasets

We analyse five different datasets that represent three popular RNA-seq library preparation methods. We started with two benchmarking datasets from the literature^2^ that sequenced five replicates of bulk mRNA using the TruSeq protocol on commercially available reference mRNAs: the Universal Human Reference RNA (UHRR; Agilent Technologies) and the Human Brain Reference RNA (HBRR, ThermoFisher Scientific). To ensure comparability, we also used UHRR aliquots to produce Smart-Seq and UMI-seq datasets in house (Table 1). However, we also wanted to include a single cell dataset, representing the most extreme and the most interesting case for low starting amounts of RNA. To this end, we chose to reanalyze the first published single cell dataset from Wu *et al.*^20^ that sequenced the cancer cell line HCT116. The library preparation method used for the single cell data is also Smart-Seq and thus comparable to our UHRR-Smart-Seq data. The only drawback that we have to keep in mind for this dataset, is that it also contains true biological variation that we cannot control for, whereas the bulk datasets using the reference mRNAs should only show technical variation.

All datasets contain ERCC-spike-ins, which allows us to compare the accuracy of the quantification of RNA-levels. Furthermore, all datasets except the UHRR-UMI-seq have paired-end sequencing, which should provide more information for the computational identification of PCR duplicates.

### Natural duplicates are expected to be common

**Figure 2:**
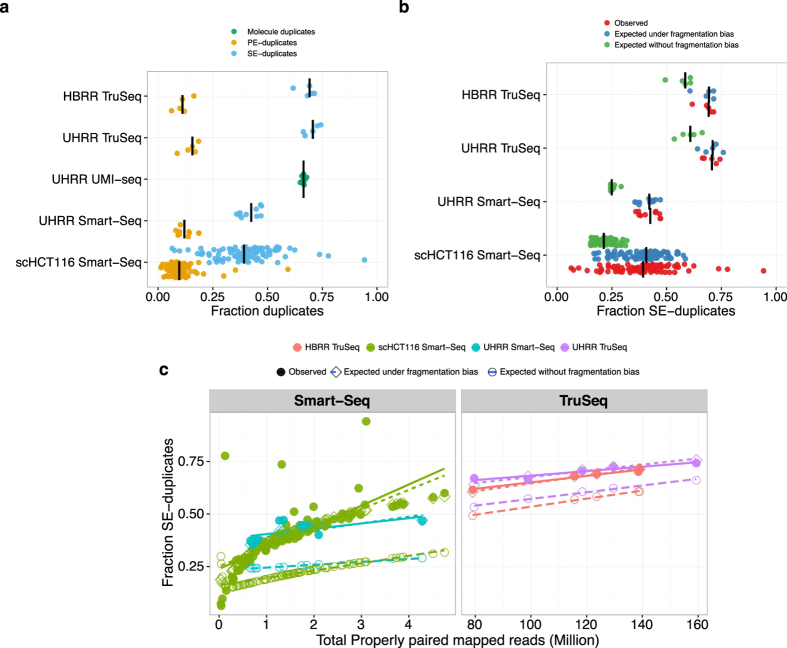
The Fraction of SE-duplicates increases with the total number of reads. In panel (**a**), we plot the fraction of computationally identified SE-duplicates (blue) and PE-duplicates (yellow) per sample. For the UMI-seq data, we identify duplicates only based on the experimental evidence provided by the UMIs. The black line marks the median for each dataset. If the correlation between sequencing depth and duplicates is due to sampling and fragmentation, we can quantify this impact. In (**b**), we plot the observed SE-duplicate fractions (red) and expected fractions (sampling–green, sampling +  fragmentation–blue). (**c**) The left panel shows the two Smart-Seq datasets (UHRR- blue, scHCT116- green) and the right panel the TruSeq data (HBRR- red, UHRR- purple). Filled circles represent the observed fraction of SE-duplicates. Open symbols represent simulated data: Open diamonds mark the expected fractions of SE-duplicates under a simple sampling model and open circles are the expectations for a sampling model with fragmentation bias. The lines are the log-linear fits between sampling depth and SE-duplicates per dataset.

The number of computationally identified paired-end read duplicates (PE-duplicates) varies between 6% and 19% for the bulk data and 1% and 59% for the single cell data. Since single-end data is commonly used for gene expression quantification, we also consider the mapping of the first read of every pair. The resulting fractions of computationally identified duplicates from single-end reads (SE-duplicates) are much higher. For the bulk data, it ranges from 36–74% and for the single cell data from 6–94% (Table 2, Fig. 2a). Surprisingly, out of the bulk datasets, the UMI-seq data show on average the highest duplicate fractions with 66% (Range: 64–68%), whereas all those duplicates are bona-fide PCR-duplicates. In the UHRR Smart-Seq data, which is the most similar dataset to the UMI-seq data, we only identified 12% PE-duplicates computationally (Fig. 2a). Although these numbers are not strictly comparable due to some differences in the library preparation (e.g. 5 more PCR-cycles for the UMI-data see Table 1 and a stronger 3′ bias (Supplementary Figure S1)), it nevertheless strongly indicates that many PCR-duplicates in Smart-Seq libraries occur during pre-amplification and thus cannot be detected by computational means.

Generally, the fraction of read duplicates is expected to depend on library complexity, fragmentation method and sequencing depth. Sequencing depth is the factor that gives us the most straight-forward predictions and in the case of SE-duplicates they are by in large independent of other parameters such as the fragment size distribution. As expected, we observe a positive correlation between the number of reads that were sequenced and the fraction of SE-duplicates (Fig. 2b,c). In order to test to what extent simple sampling can explain the number of SE-duplicates, we calculate the expected fraction of SE-duplicates, given the observed number of reads per gene and the gene lengths (see Methods, Fig. 2b,c). Note that in the case of Smart-Seq this approach will only evaluate the effect of the library PCR, but be oblivious to PCR duplicates that arose during pre-amplification. We find that for TruSeq and Smart-Seq the majority of SE-duplicates are expected under this simple model of random sampling (Fig. 2b,c). For the TruSeq data our simple model underestimates the fraction of duplicates on average by 10% (8.1–13.6%), for the single cell Smart-Seq data by 19% (0.3–67%) and for the bulk Smart-Seq data by 16.6% (11.5–22.3%). Thus, irrespective of the library preparation protocol a large fraction of computationally identified SE-duplicates could easily be natural duplicates (Fig. 2b,c).

**Figure 3:**
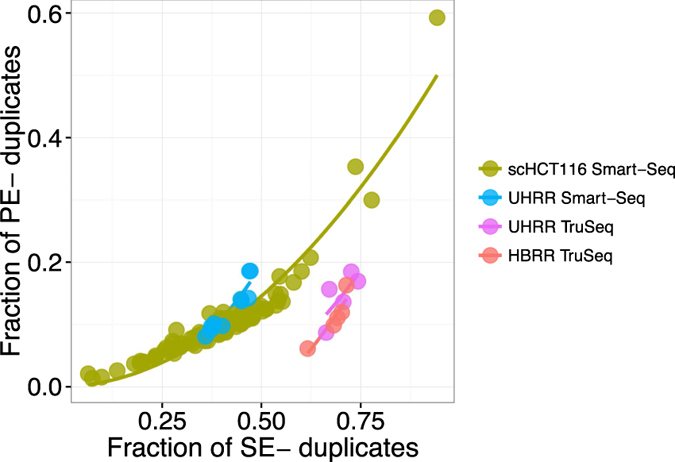
The relation between SE- and PE-duplicates. The relation between SE- and PE-duplicates is expected to follow a quadratic function, if the majority of duplicates are natural, i.e. due to fragmentation and sampling. Here, we show a quadratic fit for the different datasets (UHRR-TruSeq–purple, HBRR-TruSeq–red, UHRR-Smart-Seq–blue, scHCT116–green).

In contrast to this simple sampling expectation for SE-duplicates, fragments produced during PCR-amplification after adapter ligation, will necessarily produce fragments with the same 5′ and 3′ end and consequently will have identical mapping for both ends. If the sampling was shallow enough so that we would not expect to draw the same 5′ end twice by chance, the 3′ end position should also be identical and no reads with only one matching 5′ end are expected. If same 5′ ends are more frequent due to biased fragmentation, we expect a higher ratio of SE- to PE-duplicates. Thus, the relationship between PE- and SE-duplicates contains information about the relative amounts of duplicates produced by fragmentation as compared to amplification. More specifically, we expect that the fragmentation component of the PE- vs. SE-duplicates should be captured by a quadratic fit with an intercept of zero (Fig. 3).

The only dataset for which the quadratic term is not significant is the UHRR-TruSeq dataset. This could be seen as an indication of a higher proportion of PCR-duplicates, but it is more likely due to the low sample size of only 5 replicates. More importantly, the quadratic term is significant and positive for the HBRR TruSeq, the UHRR Smart-Seq and the scHCT116 datasets, supporting the notion that at least for those datasets library PCR amplification is not the dominant source of duplicates. This is also consistent with our finding that most observed SE-duplicates are simply due to sampling (Supplementary Table S1 and Fig. 3).

### Fragmentation is biased

**Figure 4:**
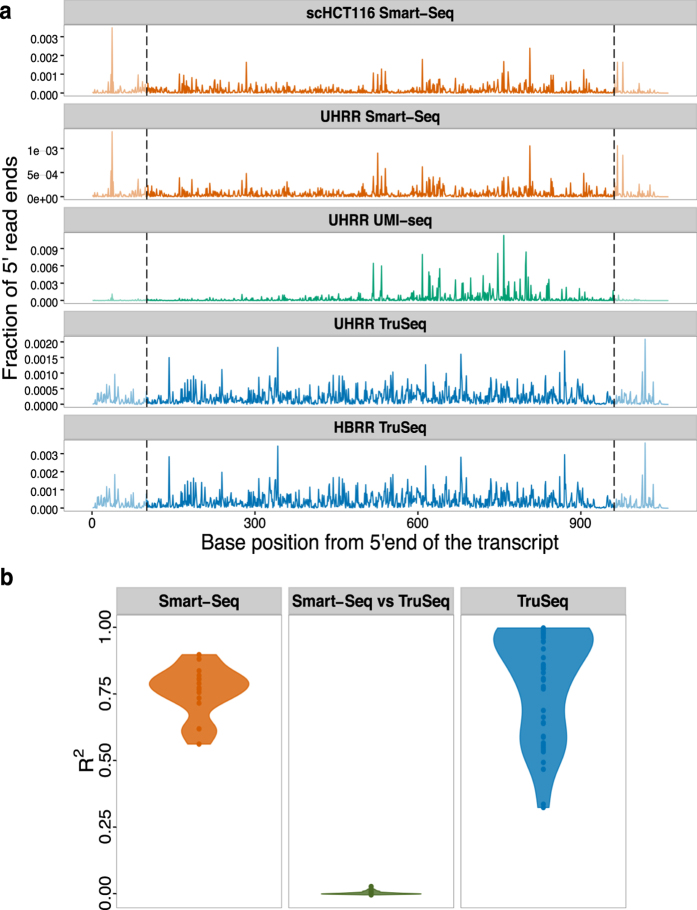
The fragmentation patterns of the ERCCs are highly reproducible for different samples prepared with the same RNA-seq library method. (**a**) Here, we plot the fraction of 5′ read ends per position of ERCC-00002. Because the TruSeq libries (blue) had read lengths of 100 bases, we do not consider the ends (grey dashed lines) for the calculation of the pair-wise *R*^2^ values. Also, note that UMI-seq creates a stronger 3′ bias. (**b**) Violin plot of the adjusted *R*^2^ of a linear model of 5′ read ends from different samples. The reproducibility of fragmentation is highest between Smart-Seq samples (orange), a little lower between the TruSeq samples and there is no correlation between samples from one Smart-Seq and one TruSeq sample (middle, green).

The model above assumes that fragmentation does occur randomly. However, some sites are more likely to break than others and this might increase the fraction of SE-duplicates. To evaluate the impact and nature of fragmentation bias, we analysed ERCC spike-ins because they are exactly the same in all datasets. First, we test whether the variance in the frequency of 5′ end mapping positions of ERCCs in one sample can explain a significant part of this variance in other samples prepared with the same method. On average, we find *R*^2^s of 0.77 and 0.85 for the Smart-Seq and TruSeq protocols, respectively. Note, that this high *R*^2^ holds for samples that were prepared in different labs: for example the *R*^2^ between the Smart-Seq samples prepared in our lab and the single cell data from the Quake lab ranges between 0.56–0.90. In contrast, if the *R*^2^ is calculated for the comparison between one TruSeq and one Smart-Seq library, it drops to 0.0012 (Fig. 4a,b). Because the UMI-seq method specifically enriches for reads close to the 3′ end of the transcript, we cannot compare fragmentation across the entire length of the transcript. However, if we limit ourselves to the 600 most 3′ basepairs, we still find that the fragmentation pattern of the UMI-seq data shows a higher concordance with the two other datasets prepared also using the Smart-Seq protocol (mean *R*^2^ =  0.08) than with the TruSeq data (mean *R*^2^ =  0.002; Supplementary Figure S2). All in all, this is strong evidence that fragmentation reproducibly prefers the same sites given a library preparation protocol and thus read sampling is not random.

To identify potential causes for these non-random fragmentation patterns, we correlated the GC-content of the 15 bases around a given position with the number of 5′ read ends. This explained very little of the fragmentation patterns in the TruSeq-data (median *R*^2^ =  0.0064, 59% of the pair-wise comparisons significant with *p* <  0.05), and none in the Smart-Seq data (median *R*^2^ =  0.00002, 18% significant with *p* <  0.05, Supplementary Figure S3a and Supplementary Table S2). Next, we built a binding motif for the Transposase^21^ from our UHRR-Smart-Seq data and, unsurprisingly, found that the motif has a very low information content (Supplementary Figure S3b) and accordingly a weak effect on the 5′ read end count (median *R*^2^ =  0.0019, 48% & 58% significant with *p* <  0.05 for scHCT116 & UHRR Smart-Seq, Supplementary Figure S3a and Supplementary Table S2).

Although we could not identify the cause for the fragmentation bias in the sequence patterns around the fragmentation site, we can still quantify the maximal impact of fragmentation bias on the number of SE-duplicates, simply by adjusting the effective length of the transcripts. For the TruSeq data, we estimate that a fragmentation bias that reduces the effective length by ~2-fold gives a reasonably good fit, leaving on average 1% (0.1–3.0%) of the SE-duplicates unexplained. For the UHRR-Smart-Seq data, a ~38.5-fold reduction in the effective length is needed and leaves only 3% (0.6–5.1%) of the duplicates unexplained. For the single cell data, the fragmentation bias that gives overall the best fit is a ~8-fold reduction, however the fit is worse since the fraction of unexplained duplicates is still at ~7% and varies between 0.3% and 61% (Fig. 2b,c). In summary, we find that fragmentation bias contributes considerably to computationally identified read duplicates and is stronger for Smart-Seq, i.e. for enzymatic fragmentation, than for TruSeq, i.e. heat fragmentation.

### Removal of duplicates does not improve the accuracy of quantification

**Figure 5:**
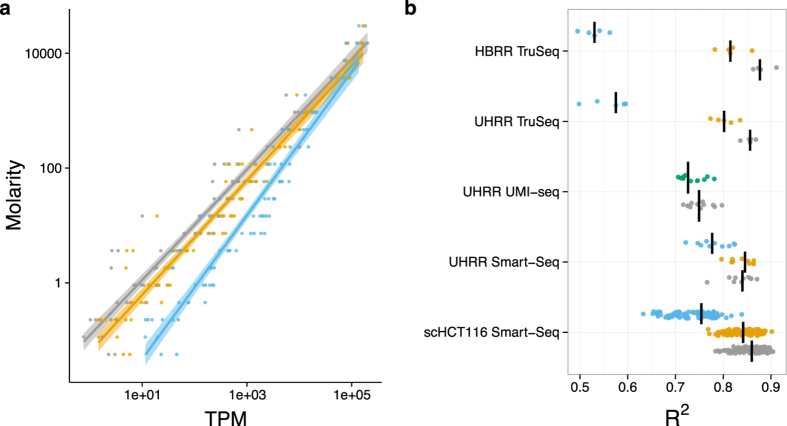
Removing duplicates does not improve the accuracy of expression quantification as measured using the ERCC spike-ins. Expression levels as quantified in transcripts per million reads (TPM) are a good predictor of the concentrations of the ERCC spike-ins. The log-linear fit of TPM vs. Molarity for one exemplary sample of the UHRR-TruSeq dataset is shown in (**a**). The most accurate prediction of ERCC molarity is the TPM estimator using all reads (grey). Removing duplicates as PE (yellow) makes the fit a little worse and removing SE-duplicates (blue) much worse. The adjusted *R*^2^ for all samples are summarized in (**b**), the median for each dataset is marked as black line. The *R*^2^ of the TPM estimate from the removal of PCR-duplicates using UMIs (green) is surprisingly similar to keeping PCR-duplicates (grey).

To evaluate the impact of PCR duplicates on the accuracy of transcript quantification, we use again the ERCC spike-in mRNAs. Although, the absolute amounts of ERCC-spike ins might vary due to handling, the relative abundances of these 92 reference mRNAs can serve as a standard for quantification. Ideally, the known concentrations of the ERCCs should explain the complete variance in read counts and any deviations are a sign of measurement errors. We calculate the *R*^2^ values of a log-linear fit of transcripts per million (TPM) versus ERCC concentration to quantify how well TPM estimates molecular concentrations and compare the fit among the different duplicate treatments. In no instance does removing read duplicates improve the fit, but in most cases the fit gets significantly worse (t-test, *p* <  2 ×  10^−3^) except for the computational PE-duplicate removal of the UHRR-Smart-Seq and the duplicate removal using UMIs (Fig. 5). These results also hold when we use a more complex linear model including ERCC-length and GC-content (Supplementary Figure S4).

### Removal of duplicates does not improve power

Most of the time we are not interested in absolute quantification, but are content to find relative differences, i.e. differentially expressed (DE) genes between groups of samples. The extra noise from the PCR-amplification has the potential to create false positives as well as to obscure truly DE genes. In order to assess the impact of duplicates on the power and the false discovery rate (FDR) to detect DE genes, we simulated data based on the estimated gene expression distributions of the five datasets. For comparability, we first equalized the sampling depth by reducing the number of mapped reads to 3 million and 1 million for bulk and single cell data, respectively. Next, we estimated gene-wise base mean expression and dispersion using DESeq2^22^.

**Figure 6:**
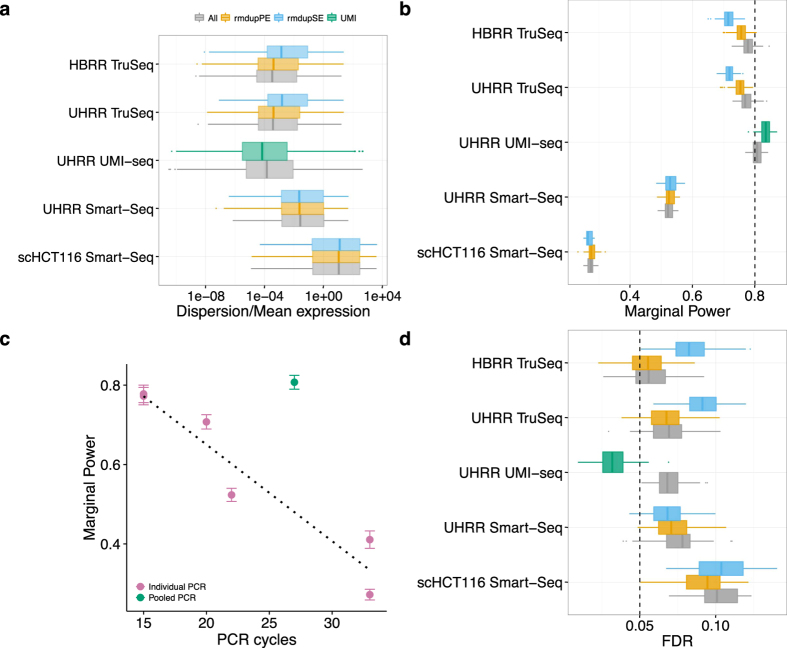
Duplicate removal has little influence on the power and FDR to detect DE-genes in comparison to the library preparation method. We estimated the distributions of mean expression and dispersion across genes for each dataset using DESeq2 after downsampling the datasets to 3 or 1 million reads. The distributions are estimated for the data including all reads (grey), removing PE-duplicates (yellow), removing SE-duplicates (blue) and for the UHRR-UMI-seq dataset removing duplicates using UMIs (green). We summarize distributions of dispersion/mean in (**a**). The estimated mean and dispersion distributions served as input for our power simulations using PROPER^19^. We did 100 simulations per dataset, whereas each dataset had two groups of six replicates (45 for scHT116) with 5% of the genes being differentially expressed between groups. In panel (**b**), we report the marginal power to detect a log2-fold change of 0.5 and in panel (**d**) the corresponding FDR, whereas the nominal FDR was set to *α* =  0.05 (dashed line). In panel (**c**), we plot our estimates of the marginal power against the number of PCR-cycles for each dataset. Error bars are standard deviation to the mean marginal power over 100 simulations. We find a surprisingly simple linear decline in power with the number of PCR-cycles, if we only consider datasets where PCR amplification was done separately for each sample of the dataset (violet). To confirm this simple fit we added two other datasets: (1) Bulk Smart-Seq dataset of mouse brain bulk RNA amplified using 20 PCR-cycles and (2) Single cell Smart-Seq dataset of 96 mouse embryonic stem cells that were amplified using 33 cycles. The only outlier is the UMI-seq dataset for which samples were pooled prior to amplification (green).

There are no big differences in the distributions of mean baseline expression and dispersion estimates from the different duplicate treatments for the two Smart-Seq datasets, whereas there is a shift towards lower means and higher dispersions, when removing SE-duplicates for the TruSeq datasets. Dispersions shift only to lower values if we exclude duplicates based on identification by UMIs (Fig. 6a, Supplementary Figure S5). The empirical mean and dispersion distributions are then used to simulate two groups with six replicates for bulk-RNA-seq datasets and 45 replicates for the single cell dataset. In all cases we simulate that 5% of the genes are differentially expressed with log2-fold changes drawn from a normal distribution with *N* (0, 1.5)^19^. We analysed 100 simulations per data-set using DESeq2 and calculate FDR and power for detecting DE-genes with a log 2-fold change of at least 0.5.

Except for the UHRR-UMI-seq dataset, the nominal FDR that we set to *α* =  5% is exceeded: the means vary between 5.4% and 10.1%, whereas the HBRR TruSeq has the lowest and the scHCT116 Smart-Seq data the highest FDR (Fig. 6d). Computational removal of SE-duplicates increases the FDR by ~2% in the HBRR-TruSeq and the UHRR-TruSeq, has no significant impact on the scHCT116 dataset and, surprisingly, improves the FDR by 1% in the UHRR-Smart-Seq data (Fig. 6d). The computational removal of PE-duplicates harbors less potential for harm, in that it leaves the FDR unchanged for both TruSeq datasets and even slightly improves the FDR for the Smart-Seq datasets. Again, the only substantial improvement is achieved by duplicate removal using UMIs, which reduces the FDR from 7% to 3%. (t-test, *p* <  1 ×  10^−15^).

The differences in the power are more striking. As for the FDR, the major differences are not between duplicate treatments, but between the datasets. For the TruSeq and the UHRR-UMI-seq datasets, the average power to detect a log2-fold change of 0.5 is ~80% (Fig. 6b). For those datasets the changes in power due to duplicate removal are only marginal and for the computational removal using PE-duplicates it actually decreases the power for the TruSeq datasets by 2%, while for the UMI-seq data duplicate removal increases power by 2%. The power for the UHRR-Smart-Seq and the scHCT116 Smart-Seq datasets is much lower with 52% and 27%, respectively, and duplicate removal increases the power by only 1%.

The large differences in power between the datasets are unlikely to be ameliorated by increasing the number of replicates per group. In addition to the 6 and 45 replicates for which the results are reported above, we also conducted simulations for 12 and 90 replicates for bulk and the single cell data, respectively. This doubling in replicate number increases the power for the UHRR-Smart-Seq dataset only from 52 to 63% and for the single cell dataset from 27 to 34% (Supplementary Figure S6, Supplementary Table 3).

## Discussion

RNA-seq has become a standard method for gene expression quantification and in most cases the sequencing library preparation involves amplification steps. Ideally, we would like to count the number of RNA molecules in the sample and thus would want to keep only one read per molecule. A common strategy applied for amplification correction in SNP-calling and ChIP-Seq protocols^23^,^24^ is to simply remove reads based on their 5′ ends, so called read duplicates. Here, we show that this strategy is not suitable for RNA-seq data, because the majority of such SE-duplicates is likely due to sampling. For highly transcribed genes, it is simply unavoidable that multiple reads have the same 5′ end, also if they originated from different RNA-molecules. We find that only ~10% (TruSeq) and ~20% (Smart-Seq) of the read duplicates cannot be explained by a simple sampling model with random fragmentation. This fraction decreases even more, if we factor in that the fragmentation of mRNA or cDNA during library preparation is clearly non-random, as evidenced by a strong correlation between the 5′ read positions of the ERCC-spike-ins across samples. Because local sequence content has little or no detectable effect on fragmentation, we cannot predict fragmentation, but we can quantify the observed effect. For example, we find that a fragmentation bias that halves the number of break points can fit the observed proportion of duplicates for TruSeq libraries well. For the Smart-Seq datasets, fragmentation biases would have to be much higher to explain the observed numbers of read duplicates. Furthermore, the fit between model estimates and the observed duplicate fractions is worse than for the TruSeq data and the model estimates for fragmentation bias are also inconsistent between the datasets (38.5 for the UHRR and 8 for the scHCT116).

Since computational methods cannot distinguish between fragmentation and PCR duplicates, the removal of read duplicates could introduce a bias rather than removing it. Using the ERCC-spike-ins, we can indeed show that removing duplicates computationally does not improve a fit to the known concentrations, but rather makes it worse, especially if only single-end reads are available (Fig. 5). This is in line with our observation that most single end duplicates are due to sampling and fragmentation. Hence, removing duplicates is similar to a saturation effect known for microarrays^25^,^26^,^27^.

Moreover, the Smart-Seq protocol, which was designed for small starting amounts, involves PCR amplification before the final fragmentation of the sequencing library. Thus in the case of Smart-Seq, computational methods cannot identify PCR duplicates that occur during the pre-amplification step. When we use unique molecular identifiers (UMIs), we find that 66% of the reads are PCR duplicates and only 34% originate from independent mRNA molecules. In contrast, when using paired-end mapping for a comparable Smart-Seq library, we identify 13% as duplicates and 87% as unique. This might in part be due to the fact that in UMI-Seq we sequence mainly 3′ ends of transcripts, thus decreasing the complexity of the library, which in turn increases the potential for PCR duplicates for a given sequencing depth (Fig. 4a, Supplementary Figure S1). However, it is unlikely that library complexity can explain the 53% difference in duplicate occurrence. This difference is more likely to be due to PCR-duplicates that are generated during pre-amplification and thus remain undetectable by computational means.

All in all, computational methods are limited when it comes to removing PCR-duplicates, but how much noise or bias do PCR duplicates introduce? In other words, we want to know how PCR-duplicates impact the power and the false discovery rate for the detection of differentially expressed genes. Both, power and FDR, are determined by the gene-wise mean expression and dispersion. Based on simulated differential expression using the empirically determined mean and dispersion distributions, we find that computational removal of duplicates has either a negligible or a negative impact on FDR and power, and we therefore recommend not to remove read duplicates. In contrast, if PCR duplicates are removed using UMIs, both FDR and power improve. Even though the effects in the bulk data analysed here are relatively small: FDR is improved by 4% and the power by 2%, UMIs will become more important when using smaller amounts of starting material as it is the case for single-cell RNA-seq^6^,^28^.

The major differences in power are between the datasets with the TruSeq and the UMI-seq data achieving a power of around 80%, the UHRR-Smart-Seq 52% and the single cell Smart-Seq data (scHCT116) only 27%. Note that this apparently bad performance of the single cell Smart-Seq data is at least in part due to an unfair comparison. While all the other datasets were produced using commercially available mRNA and thus represent true technical replicates, the single cell data necessarily represent biological replicates and thus are expected to have a larger inherent variance and thus lower power.

However, also the UHRR Smart-Seq bulk data achieves with 52% a much lower power than the other bulk datasets. One possible explanation for the differences in power is the total number of PCR-cycles involved in the library preparation. With every PCR-cycle the power to detect a log 2-fold change of 0.5 appears to drop by 2.4% (Fig. 6c). The only exception is the UMI-seq dataset, that gives a power of 81%, even if duplicates are not removed, which is comparable to the power reached with TruSeq data despite the UMI-seq method having 12 more PCR-cycles. Technically UMI-seq is most similar to the Smart-Seq method. The biggest difference between the two methods is that all UMI-seq libraries are pooled before PCR-amplification, suggesting that the PCR-noise is due to the different PCR-reactions and not due to amplification efficiency per-se.

We conclude that computational removal of duplicates is not recommendable for differential expression analysis and if sufficient starting material is available so that only few PCR-cycles are necessary, the loss in power due to PCR duplicates is negligible. However, if more amplification is needed, power would be improved if all samples are pooled early on, and for really low amounts as for single cell data also the gain in power that is achieved by removing PCR-duplicates using UMIs will become important.

## Methods

### Datasets

We used six datasets representing the TruSeq, Smart-Seq and UMI-seq protocols and varying amounts of starting material from bulk RNA or single cell RNA. All analysed datasets contain the ERCCs spike-in RNAs. This is a set of 92 artifical poly-adenylated RNAs designed to match the characteristics of naturally occurring RNAs with respect to their length (273–2022 bp), their GC-content (31–53%) and concentrations of the ERCCs (0.01–30,000 attomol/*μl*). The recommended ERCC spike-in amounts result in 5–10^7^ ERCC RNA molecules in the cDNA synthesis reaction.

To reduce biological variation, we used the well-characterized Universal Human Reference RNA (UHRR; Agilent Technologies) for the two datasets produced for this study. We downloaded UHRR- and HBRR-TruSeq data from SEQC/MAQC-III^2^. Finally, we also analyse the single cell data published in Wu *et al.*^20^, for which the colorectal cancer cell-line HCT116 was used (Table 1). The input mostly being commercially distributed human samples, we expect all biological samples analysed in this study to have similarly high quality and complexity. All data that were generated for this project were submitted to GEO under accession GSE75823.

### RNA-seq library preparation and sequencing

For the Smart-Seq libraries, 250 ng of Universal Human Reference RNA (UHRR; Agilent Technologies) and ERCC spike-in control mix I (Life Technologies) were used and cDNA was synthesized as described in the Smart-Seq2 protocol from Picelli *et al.*^13^. However, because we used more mRNA to begin with, we reduced the number of pre-amplification PCR cycles to 9 cycles instead of the 18–21 recommended in Picelli *et al.*^13^. 1 ng of pre-amplified cDNA was then used as input for Tn5 transposon tagmentation by the Nextera XT Kit (Illumina), followed by 12 PCR cycles of library amplification. For sequencing, equal amounts of all libraries were pooled.

For the UMI-seq libraries, we started with 10 ng of UHRR-RNA to synthesise cDNA as described in Soumillon *et al.*^16^. This protocol is very similar to the Smart-Seq protocol, however the first strand cDNA is decorated with sample-specific barcodes and unique molecular identifiers. The barcoded cDNA from all samples was then pooled, purified and unincorporated primers digested with Exonuclease I (NEB). Pre-amplification was performed by single-primer PCR for 15 cycles. 1 ng of full-length cDNA was then used as input for the Nextera XT library preparation with the modification of adding a custom i5 primer to enrich for barcoded 3′ ends.

Library pools were sequenced on an Illumina HiSeq1500. The Smart-Seq libraries were sequenced using 50 cycles of paired-end sequencing on a High-Output flow-cell. The UMI-seq libraries were sequenced on a rapid flow-cell with paired-end layout, where the first read contains the sequences of the sample barcode and the UMI sequence using 17 cycles. The second read contains the actual cDNA fragment with 46 cycles.

### Data Processing

For Smart-Seq and TruSeq libraries, the sequenced reads were mapped to the human genome (hg19) and the splice site information from the ensembl annotation (GRCh37.75) using STAR(version:2.4.0.1)^29^ with the default parameters, reporting only the best hit per read. The genome index was created with –sjdbOverhang ‘readlength-1’. Because the ERCCs are transcript sequences no splice-aware mapping is neccessary and therefore we used NextGenMap for the ERCCs^30^. Except for three parameters, (1) the maximum fragment size which was set to 10 kb, (2) the minimum identity set to 90% and (3) reporting only the best hit per read, we also used the default parameters for NextGenMap. Note that we also included hg19 and did not map to ERCC sequences only. The mapped reads were assigned to genes [Ensembl database annotation version GRCh37.75] using FeatureCount from the bioconductor package Rsubread^31^ (see Supplementary text).

For UMI-seq data, cDNA reads were mapped to the transcriptome as recommended in Soumillon *et al.*^16^ using the Ensembl annotation [version GRCh37.75] and NextGenMap^30^ (Supplementary text). If either the sample barcode or the UMI had at least one base with sequence quality ≤10 or contained ‘N’s the read was discarded. Next, we generated count tables for reads or UMIs per gene. Finally, mitochondrial and ambiguously assigned reads were removed from all libraries.

### Duplicate detection and removal

We defined single-end (SE) read duplicates as reads that map to the same 5′ position, have the same strand and the same CIGAR value. Because we cannot determine the exact mapping position for 5′ soft clipped reads, we discard them. To flag paired-end duplicates (PE), we used the same requirements as for the SE-duplicates, those requirements had just to be fulfilled for both reads of a pair.

### Model for the fraction of sampling and fragmentation duplicates

We obtain an expectation for the number of reads if duplicates are identified via their 5′ position and only one read per 5′ end position is kept. The only input parameters are the observed number of reads per gene (*r*_*G*_) and the effective length of the gene (*L*_*eG*_ =  *L* −  2 ×  read-length). Then the expected number of unique reads can be estiamted as


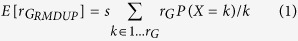


whereas *P* (*X* =  *k*) can be calculated using a positive Poisson distribution with *λ*_*G*_ =  *r*_*G*_/*L*_*eG*_ and *s* is a scaling factor 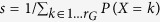.

In order to estimate the level of fragmentation bias, we simply modified the effective length *L*_*eG*_ by a factor *f* ×  *L*_*eG*_.

### Fragmentation pattern analysis

To compare fragmentation sites across libraries, we counted 5′ read starts per position for the ERCCs across all datasets using samtools and in house perl scripts. To avoid edge effects in later analyses, we excluded the first and last 100 bases of each ERCC, whereas 100 bases is the maximum read length of datasets analysed here.

We generated a Position Weight Matrix (PWM) for the transposase (Tn5) motif by simply stacking up the 30 bases of the putative Transposase binding sites from all UHRR-Smart-Seq reads. Those 30 bases are identified as 6 bases upstream of the 5′ read end and the 24 downstream^21^. The resulting PWM was then used to calculate motif scores across the ERCCs using the Bioconductor package PWMEnrich^32^.

### Power evaluation for differential expression

For power analysis, we estimated the mean baseline expression and dispersion for all datasets after downsampling them to 3 and 1 million reads for bulk and single cell data, respectively. This was done for all three duplicate treatments (keep all, remove SE and remove PE) using DESeq2^22^ with standard parameters. Furthermore, genes with very low dispersions (< 0.001) were removed. We chose the sample sizes 3, 6 and 12 per condition for the bulk data and 30, 45 and 90 for the single cell dataset, because they seemed to be a good representation of the current literature. For the simulations, we use an in-house adaptation of the Bioconductor-package PROPER^19^. As suggested in Wu *et al.*^19^, we set the fraction of differentially expressed genes between groups to 0.05 and the log2-fold change for the DE-genes was drawn from a normal distribution with *N* (0, 1.5). We generated 100 simulations per original input data-set and analysed them using DESeq2. Next, we calculated the power to detect a log2-fold change of at least 0.5 and the according FDR using *α* =  0.05.

## Additional Information

**Accession codes**: RNA-seq data generated for this study is submitted to GEO under the accession code: GSE75823.

**How to cite this article**: Parekh, S. *et al.* The impact of amplification on differential expression analyses by RNA-seq. *Sci. Rep.* **6**, 25533; doi: 10.1038/srep25533 (2016).

## Supplementary Material

Supplementary Information

## Figures and Tables

**Figure 1:**
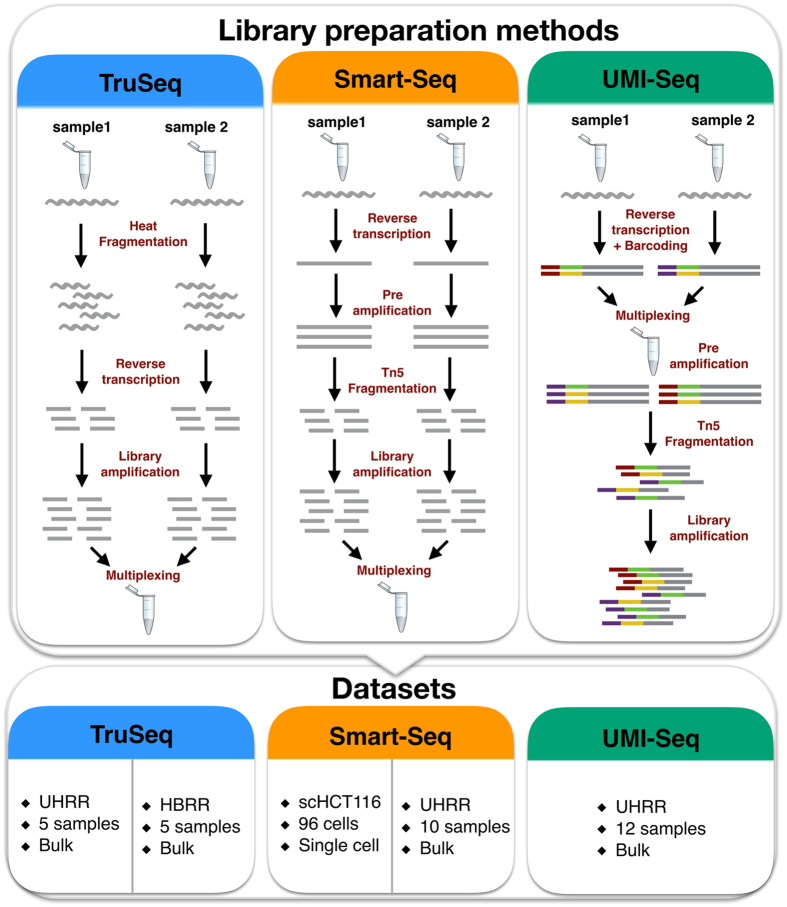
Schematic of library preparation protocols and datasets. The upper panel details the steps for the three sequencing library preparation methods analysed in this study. In the UMI-seq flow-chart red and purple tags represent the sample barcodes and the green and yellow tags the UMIs.

**Table 1:** Description of the datasets analysed.

Study ID	GSE-ID	Lab	Sample size	Reads per sample (Mean ± SD million)	Read Length	PCR cycles
scHCT116 Smart-Seq	GSE51254	Quake	96	1.8 ± 1.1	101	21* + 12
UHRR Smart-Seq	GSE75823	Enard	10	1.5 ± 1.1	50	10* + 12
UHRR UMI-seq	GSE75823	Enard	12	9 ± 1	46	15* + 12
UHRR TruSeq	GSE49712	SEQC	5	125 ± 33	101	15
HBRR TruSeq	GSE49712	SEQC	5	140 ± 29	101	15

^*^preamplification PCR-cycles.

**Table 2:** Fraction of duplicates per sample.

Study Name	Fraction PE-duplicates	Fraction SE-duplicates
HBRR TruSeq	0.06–0.16	0.62–0.71
scHCT116 Smart-Seq	0.013–0.59	0.064–0.94
UHRR Smart-Seq	0.081–0.18	0.36–0.47
UHRR TruSeq	0.087–0.18	0.66–0.74
UHRR UMI-seq	0.65–0.68*	

^*^Fraction of duplicates based on UMI counts.
