# Supplementary material for: The impact of amplification on differential expression analyses by RNA-seq
Source: Sci Rep. 2016 May 9;6:25533. doi: 10.1038/srep25533 (PMC4860583; doi:10.1038/srep25533)
Supplement: Supplementary Information [file srep25533-s1.pdf]

The impact of amplification on differential expression analyses by RNA-seq

Swati Parekh, Christoph Ziegenhain, Beate Vieth, Wolfgang Enard, Ines Hellmann*

Anthropology & Human Genomics, Department of Biology II, Ludwig-Maximilians University, Großhaderner Str. 2, 82152 Martinsried, Germany.

* hellmann@bio.lmu.de

Supplementary figures

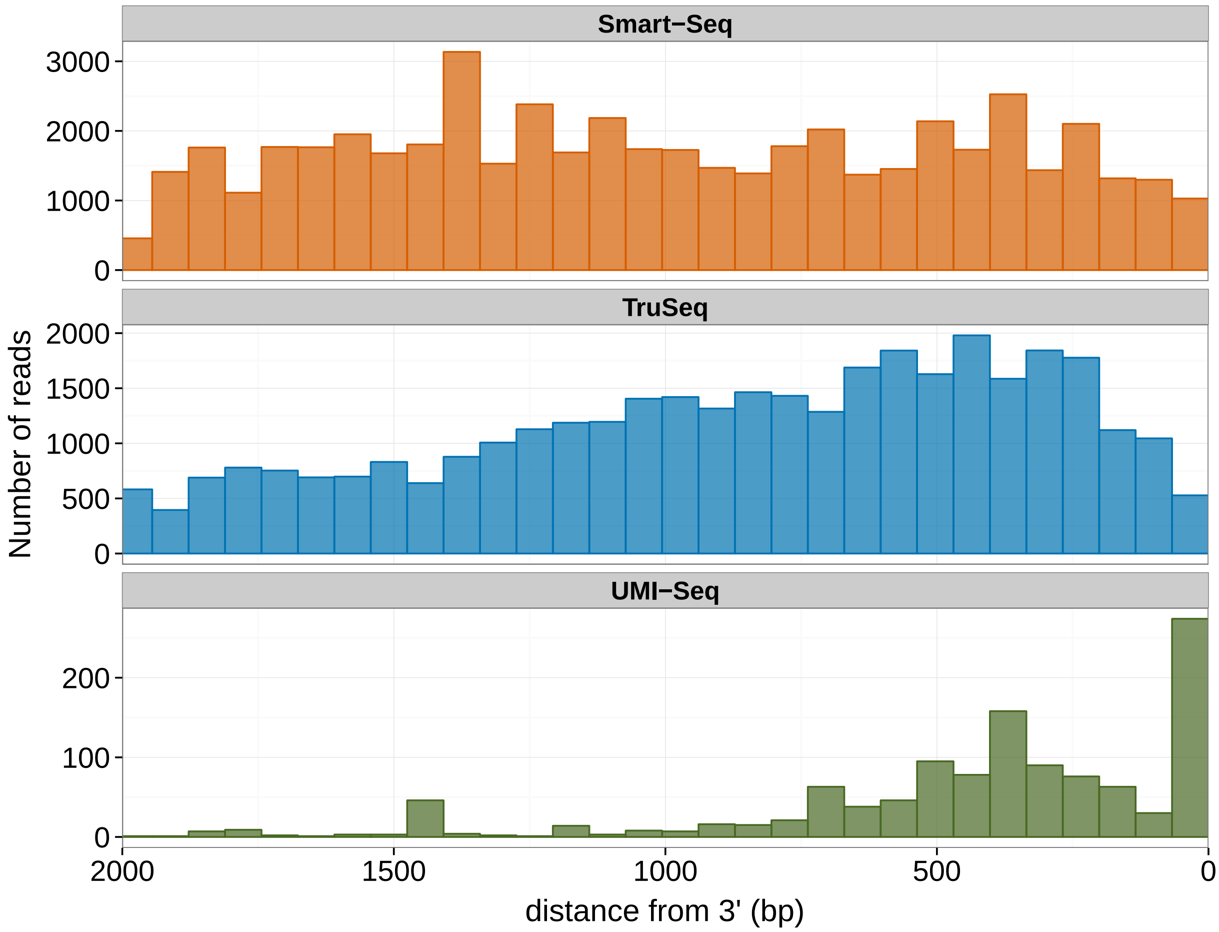

Figure S1: 3' bias in fragmentation site is prominent in UMI-seq. The histogram showing distance of the fragmentation site from 3' end of the gene measured from ERCC spike-ins of length $\sim 2kb$. Colors represent library preparation methods, 'blue' - Smart-Seq, 'orange' - TruSeq, 'green' - UMI-seq.

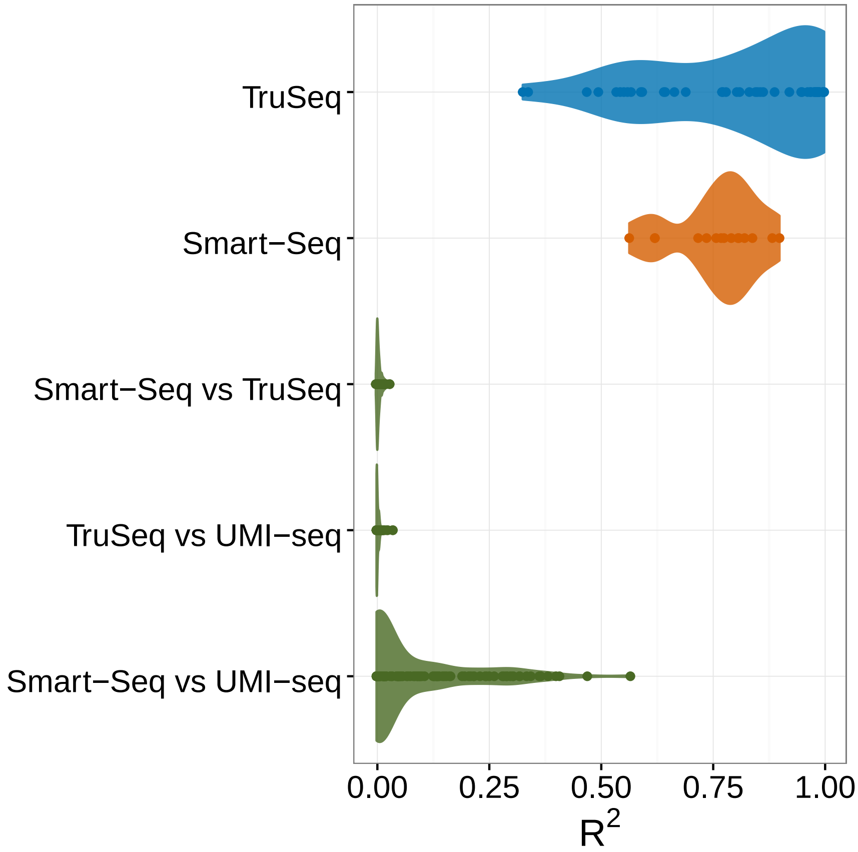

Figure S2: The fragmentation patterns of the most 3' 600bp of ERCCs are relatively reproducible between Smart-Seq and UMI-seq. Violin plots of the adjusted R^2 from a linear model between fraction of 5'read ends from different samples. The adjusted R^2 are calculated considering full length for Smart-Seq and TruSeq methods whereas for comparison to UMI-seq the most 3' 600bp are considered. The reproducibility of fragmentation is highest within Smart-Seq (orange) and TruSeq samples (blue). Fragmentation reproducibility between Smart-Seq and UMI-seq samples(green) is higher than compared to TruSeq(green), as both methods use transposase tagmentation.

a **b**

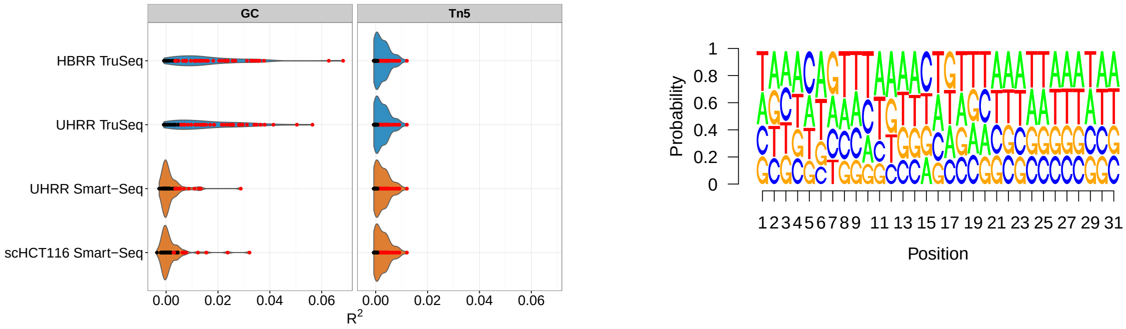

Figure S3: Fragmentation does not appear to have a cutting site preference. Colors of the violin plots represent library preparation methods, 'blue - Smart-Seq, 'orange' - TruSeq and dots are colored by the significance of the fit where 'red' - pvalue ≤ 0.05 and 'black' - pvalue > 0.05. **a)** The left panel shows violin plots of the adjusted R^2 of linear model fit between background corrected GC content of 15bases window and fraction of 5'read ends of the middle base in the window for each ERCC spike-in and the right panel shows the adjusted R^2 of linear model fit between Tn5 motif score calculated for ERCC spike-in RNAs. **b)** Sequence logo of the Tn5 motif derived from UHRR Smart-Seq dataset.

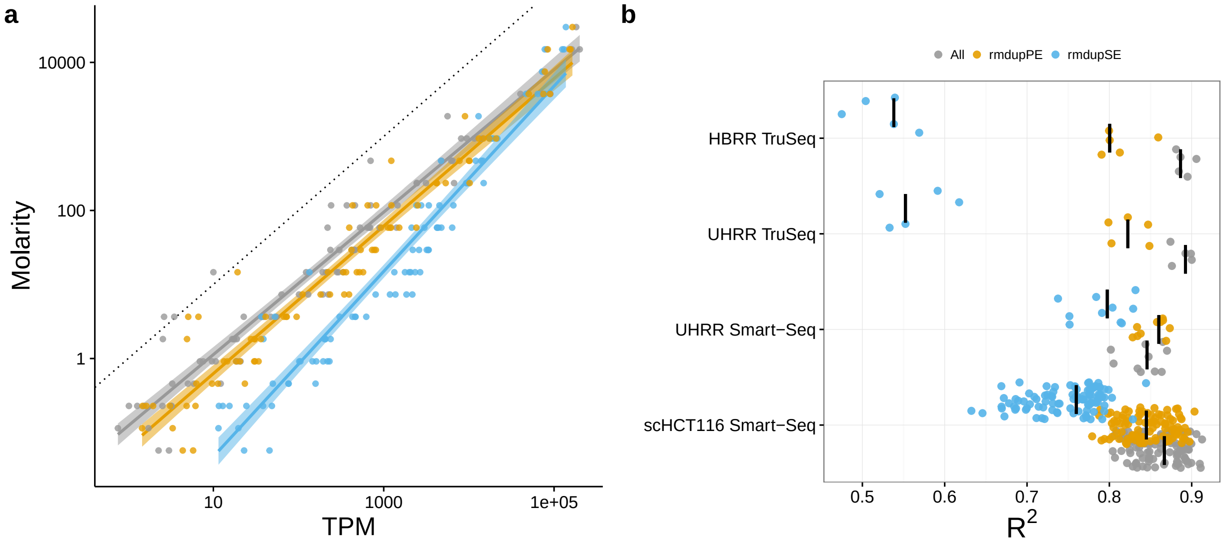

Figure S4: Removing duplicates does not improve the accuracy of expression quantification as measured using the ERCC spike-ins. Expression levels as quantified in transcripts per million reads (TPM) are considered to be good measure of ERCC spike ins. However, other factors like capture and sequencing efficiency can not be explained by TPM. One exemplary sample of the UHRR-TruSeq dataset as shown in Figure 5 of the main text is shown in **a)**. The dashed grey line shows the bisecting line. We calculated the log-linear fit of counts per million (CPM) vs. Molarity also controlling for GC content and length of the transcript. The adjusted R^2 for all samples are summarized in **b)**, the median for each dataset is marked as black line. The colors represent different duplicates treatment. All reads (grey), removing PE-duplicates (yellow) and removing SE-duplicates (blue).

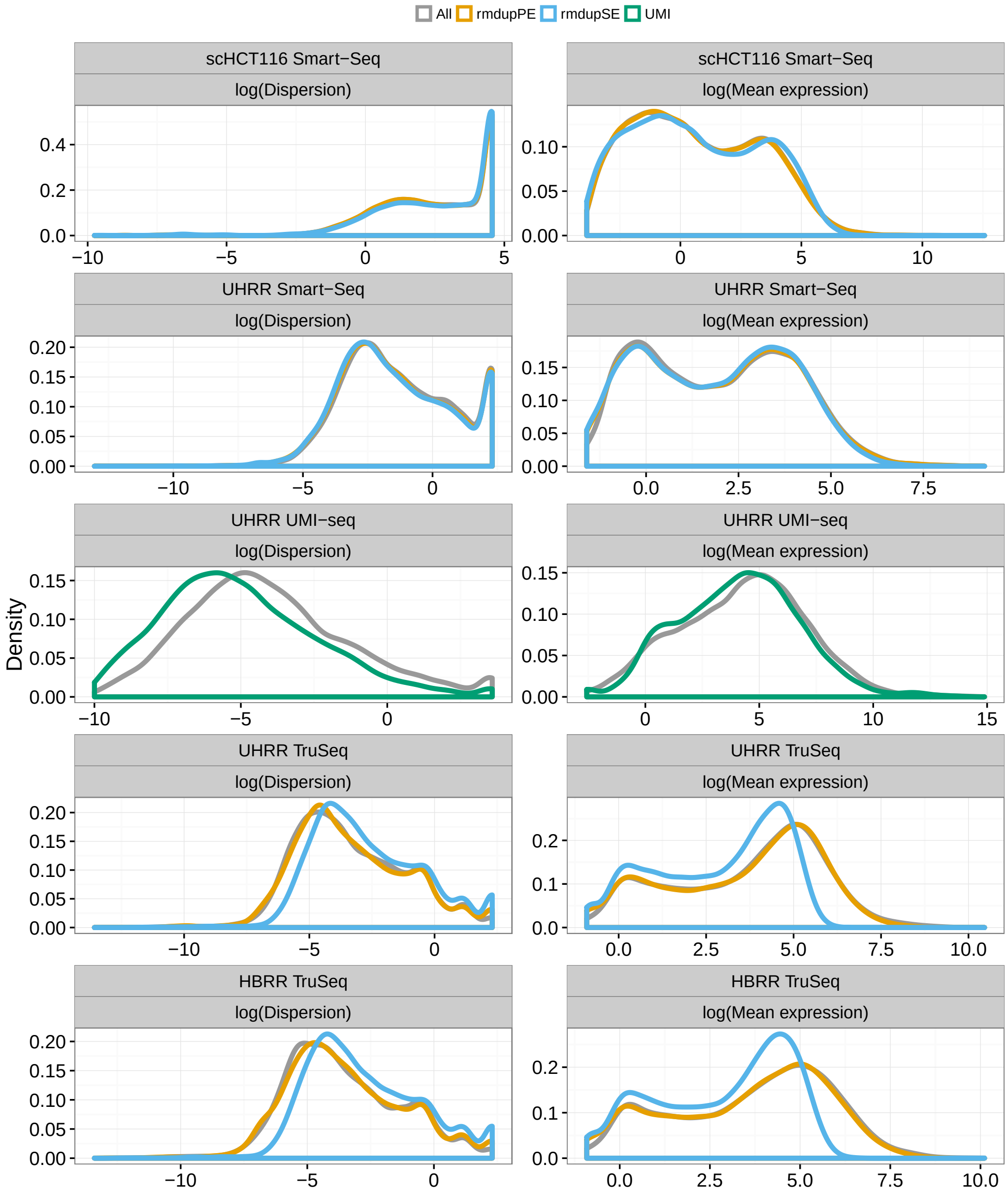

Figure S5: Empirical mean and dispersion distributions are used to estimate power to detect differential expression. The left panel shows density plot of log(dispersion) and the right panel the log(mean baseline expression) measured by DESeq2 for each study. Different duplicates treatments are represented by colors, All reads- grey, removing PE-duplicates- orange, removing SE-duplicates- blue and removing duplicate molecules in UMI-seq as green.

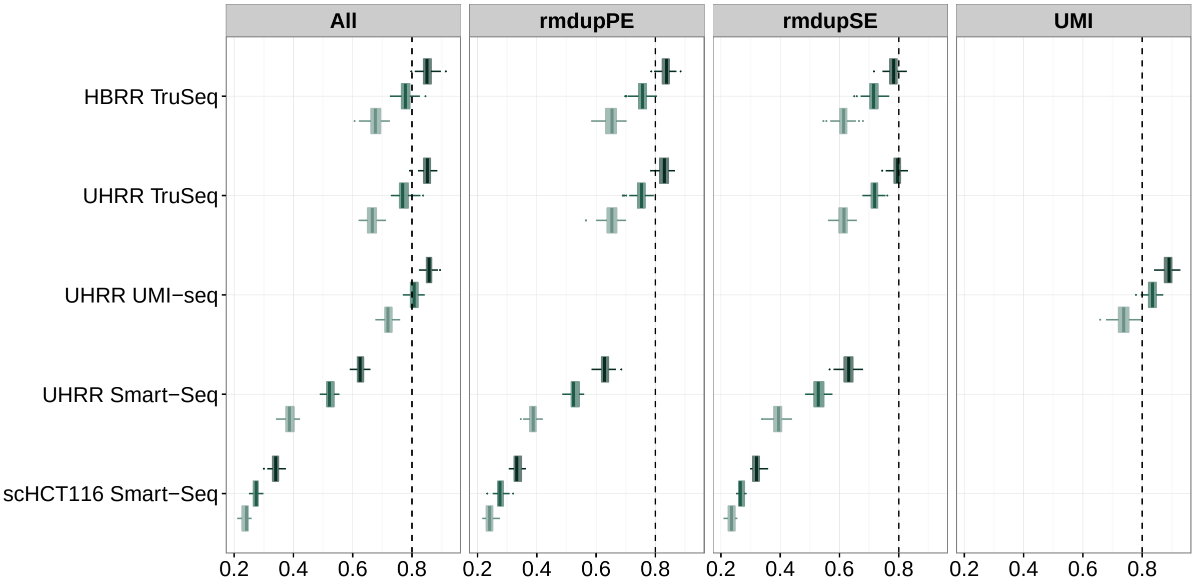

Figure S6: Power to detect differential expression increases with increased sample size. The boxplot shows marginal power to detect 0.5 log2foldchange at 5% nominal FDR for different sample sizes. Colors gradient from light to dark represent sample sizes 3,6 and 12 for the bulk and 30,45 and 90 for the single cell datasets.

Supplementary text

Detailed commands used for mapping are given below.

STAR genome generate

STAR –runThreadN 10 –runMode genomeGenerate –genomeDir hg19STARindex –genomeFastaFiles hg19.fa –sjdbGTFfile GRCh37.75.gtf –sjdbOverhang ‘readLen-1‘

STAR mapping

STAR –readFilesIn R1.fastq R2.fastq –runThreadN 10 –outFileNamePrefix samplename –outFilterMultimapNmax 1 –outSAMunmapped Within –outSAMtype BAM SortedByCoordinate –sjdbGTFfile GRCh37.75.gtf –genomeDir hg19STARindex –sjdbOverhang ‘readLen-1‘ –outFilterType BySJout –outSJfilterReads Unique

NextGenMap mapping

For ERCC spike-ins

ngm.4.12 -1 R1.fastq -2 R2.fastq -t 10 -i 0.9 -X 10000 -r ERCCs.fa -o samplename.sam

For UMI-seq data

ngm.4.12 -q R1.fastq -t 10 -i 0.9 -r GRCh37.75.fa -o samplename.sam

Supplementary tables

Table S1: Summary of squared terms from quadratic fit between PE-dup and SE-dup (PE-dup $\sim$ SE-dup+(SE-dup)^2+0)

Study name	Beta^2	Std. Error	t value	Pr($>$ |t|)
scHCT116 Smart-Seq	0.542	0.0302	17.94	0.0000
UHRR Smart-Seq	1.168	0.246	4.739	0.001
UHRR TruSeq	0.840	0.619	1.356	0.268
HBRR TruSeq	1.134	0.338	3.350	0.044

Table S2: Median R^2 and percentage of significant ERCCs for the lm fit between GC content/Tn5 motif score and 5' read ends

Study name	GC		Tn5	
	R^2	%Significant*	R^2	%Significant*
scHCT116 Smart-Seq	-0.00027	16%	0.00112	49%
UHRR Smart-Seq	0.00020	19%	0.00174	59%
UHRR TruSeq	0.00614	57%	0.00077	43%
HBRR TruSeq	0.00657	61%	0.00077	43%

*Percentage of ERCCS with p-value ≤ 0.05

Table S3: Summary of power analysis

Study name	Sample size	Mean FDR	Marginal power	Avg # of TD	Avg # of FD	FDC	DupType	PCRcycles	Amount(ug)
HBRR TruSeq	3	0.06	0.68	239.63	16.28	0.07	All	15	1.00
HBRR TruSeq	3	0.06	0.65	232.52	16.35	0.07	rmdupPE	15	1.00
HBRR TruSeq	3	0.07	0.61	266.98	20.45	0.08	rmdupSE	15	1.00
HBRR TruSeq	6	0.06	0.78	277.37	19.16	0.07	All	15	1.00
HBRR TruSeq	6	0.05	0.76	273.61	17.75	0.06	rmdupPE	15	1.00
HBRR TruSeq	6	0.08	0.72	315.48	31.46	0.10	rmdupSE	15	1.00
HBRR TruSeq	12	0.06	0.85	307.49	21.32	0.07	All	15	1.00
HBRR TruSeq	12	0.05	0.84	298.30	19.26	0.06	rmdupPE	15	1.00
HBRR TruSeq	12	0.07	0.78	352.17	30.74	0.09	rmdupSE	15	1.00
scHCT116 Smart-Seq	30	0.14	0.24	194.30	33.80	0.17	All	33	0.00
scHCT116 Smart-Seq	30	0.14	0.24	208.35	34.00	0.16	rmdupPE	33	0.00
scHCT116 Smart-Seq	30	0.15	0.23	211.20	37.70	0.18	rmdupSE	33	0.00
scHCT116 Smart-Seq	45	0.10	0.27	230.45	26.60	0.12	All	33	0.00
scHCT116 Smart-Seq	45	0.09	0.28	246.70	25.35	0.10	rmdupPE	33	0.00
scHCT116 Smart-Seq	45	0.10	0.27	251.00	29.35	0.12	rmdupSE	33	0.00
scHCT116 Smart-Seq	90	0.06	0.34	293.92	21.13	0.07	All	33	0.00
scHCT116 Smart-Seq	90	0.07	0.33	307.00	22.35	0.07	rmdupPE	33	0.00
scHCT116 Smart-Seq	90	0.07	0.32	308.55	22.75	0.07	rmdupSE	33	0.00
UHRR UMI-seq	3	0.06	0.72	447.41	33.19	0.07	All	27	0.01
UHRR UMI-seq	3	0.03	0.74	238.36	7.00	0.03	UMI	27	0.01
UHRR UMI-seq	6	0.07	0.81	507.54	43.54	0.09	All	27	0.01
UHRR UMI-seq	6	0.03	0.83	271.73	10.30	0.04	UMI	27	0.01
UHRR UMI-seq	12	0.06	0.86	553.42	43.01	0.08	All	27	0.01
UHRR UMI-seq	12	0.04	0.89	301.07	13.42	0.04	UMI	27	0.01
UHRR Smart-Seq	3	0.06	0.39	288.66	18.89	0.07	All	22	0.25
UHRR Smart-Seq	3	0.06	0.39	282.26	17.25	0.06	rmdupPE	22	0.25
UHRR Smart-Seq	3	0.05	0.39	283.54	15.46	0.05	rmdupSE	22	0.25
UHRR Smart-Seq	6	0.08	0.52	404.17	34.57	0.09	All	22	0.25
UHRR Smart-Seq	6	0.07	0.53	399.62	32.43	0.08	rmdupPE	22	0.25
UHRR Smart-Seq	6	0.07	0.53	398.36	30.53	0.08	rmdupSE	22	0.25
UHRR Smart-Seq	12	0.06	0.63	489.58	35.81	0.07	All	22	0.25
UHRR Smart-Seq	12	0.06	0.63	483.90	34.61	0.07	rmdupPE	22	0.25
UHRR Smart-Seq	12	0.06	0.63	481.09	32.36	0.07	rmdupSE	22	0.25
UHRR TruSeq	3	0.08	0.67	274.02	25.72	0.09	All	15	1.00
UHRR TruSeq	3	0.08	0.65	269.81	25.53	0.09	rmdupPE	15	1.00
UHRR TruSeq	3	0.08	0.61	316.45	30.10	0.10	rmdupSE	15	1.00
UHRR TruSeq	6	0.07	0.77	319.40	26.78	0.08	All	15	1.00
UHRR TruSeq	6	0.07	0.75	314.12	25.36	0.08	rmdupPE	15	1.00
UHRR TruSeq	6	0.09	0.72	375.37	41.36	0.11	rmdupSE	15	1.00
UHRR TruSeq	12	0.06	0.85	350.17	24.90	0.07	All	15	1.00
UHRR TruSeq	12	0.05	0.83	345.31	22.83	0.07	rmdupPE	15	1.00
UHRR TruSeq	12	0.08	0.79	412.77	39.44	0.10	rmdupSE	15	1.00
